# Perchloride of Iron in Diphtheritis

**Published:** 1860-03

**Authors:** F. Isnard


*Perchloride of Iron in Diphtheritis.* By Dr. F. Isnard.

The following are the conclusions presented by Dr. Isnard, with which he closes his memoir on the nature and treatment of this disease:

Croup and *angina membranacea* are special inflammations of the fauces and air-passages, with a peculiar alteration of their mucous membranes, which allows fibrino-albuminous products, formed at the expense of the elements of the blood, to transude in the form of pseudo-membranes.

They are always, at the commencement, local affections. Sometimes they remain local; sometimes they are infectious. Diphtheritic infection is always consecutive, never primitive. The cause is the alteration and resorption of the pseudo-membranous products, which is analogous to the purulent resorption that is always consecutive to a solution of continuity or to any inflammatory condition. The rapidity and the importance of diphtheritic poisoning vary in accordance with a host of unknown conditions, among which the epidemic character plays a prominent part.

The false membrane, being the cause of all the grave phenomena which appear in the course of membranous affections, as much by its mechanical agency (suffocation, asphyxia, &c.) as by its dynamic effects, (resorption and diphtheritic poisoning, &c.) to prevent its formation, or to destroy it when formed, is the duty of therapeutics. The treatment is medical and surgical, or external. Rational medical treatment consists in putting the blood quickly in such a condition that its fibrino-albuminous elements cannot transude the mucous membranes, or that they shall not escape except in a form almost serous. Fluidifying and alterative agents have hitherto had the most reputation in the medical treatment of croup. But in general they act too slowly, too feebly, have the inconvenience of weakening the system, and without preventing entirely the danger of diphtheritis; hence they have been rejected. Of all these, tartar-emetic, in large doses, has produced the greatest number of cures.

Coagulating agents act more rapidly upon the blood, and have the advantage of removing none of its elements, and of preventing the ultimate accidents of membranous affections. In this class, the perchloride of iron, by its harmlessness to the system and the promptitude of its action, merits the preference. It is the sheet-anchor of therapeutics in croup, a species of specific for that terrible disease.

The action of perchloride of iron in these diseases is triple:

1. Action on the blood, whose fibrino-albuminous elements it makes more or less plastic, and makes it thus impossible for them to pass through the mucous respiratory surface; and afterwards, in infectious cases, to pass back into uriniferous tubes, solutions of cutaneous continuity, &c.

2. Action on the respiratory mucous membrane, whose fibrino-albuminous elements it plastifies, and closes up the organic tissue. In this way the mucous tissue becomes incapable of admitting the passage of the albuminoid principles of the blood.

3. Tonic action, strengthening the nervous system; an essential action, according to many physicians, but of secondary importance, in my opinion, in the treatment of croup.

The perchloride of iron should be administered as soon as possible after the inception of the disease, in large doses. Its use should be continued at all stages of the disease, both when false membranes are formed and when the general infection is established. Under all these circumstances its action will be the same; an action rather physico-chemical on the elements of the blood and the respiratory mucous membrane, than dynamical on the nervous system.

The surgical and external treatment is also important. It consists in frictions with croton oil on the neck, with revulsives to the extremities; cauterization of the false membranes at accessible points; inhalation of alkaline solutions; and, if necessary, tracheotomy.—*Gazette des Hôpitaux.* L. H. S.

